# Crystalline and Amorphous Cerium Phosphate Nanoparticles as Enzyme-Mimetic Nanomaterials for Regenerative Medicine

**DOI:** 10.3390/nano16171074

**Published:** 2026-08-29

**Authors:** Ekaterina V. Silina, Artem S. Chizhov, Maria P. Kruglova, Alexey A. Kryukov, Svetlana A. Dodonova, Maksim A. Pugachevskii, Natalia E. Manturova, Victor A. Stupin

**Affiliations:** 1 Institute of Biodesign and AI in Medicine, I.M. Sechenov First Moscow State Medical University (Sechenov University), 119991 Moscow, Russia; marykruglova@live.ru; 2 Chemistry Department, Lomonosov Moscow State University, 119991 Moscow, Russia; chizhov@inorg.chem.msu.ru; 3 Institute of Experimental Medicine, Kursk State Medical University, 305041 Kursk, Russia; krukovaa@kursksmu.net (A.A.K.); dodonovasveta@mail.ru (S.A.D.); 4 Center for Photonics & 2D Materials, Moscow Center for Advanced Studies, 123592 Moscow, Russia; maxpugachevskii@gmail.com; 5 Department of Surgery, Pirogov Russian National Research Medical University, 117997 Moscow, Russia; manturovanatali@yandex.ru (N.E.M.); stvictor@bk.ru (V.A.S.)

**Keywords:** cerium phosphate nanoparticles, enzyme-mimetic activity, redox, Ce^3+^/Ce^4+^ mixed valence, oxygen vacancies, fibroblasts, keratinocytes, biocompatibility, controlled hydrolysis, wound healing

## Abstract

Wounds represent a persistent challenge in modern medicine, driving the search for novel nanomaterials capable of modulating oxidative stress and promoting tissue regeneration. While cerium oxide nanoparticles have been extensively studied for their enzyme-mimetic activity, hydrated cerium orthophosphates remain relatively unexplored despite their promising biocompatibility and redox properties. The aim of the study is the synthesis of cerium phosphate nanoparticles (CePO_4_ NPs) via controlled hydrolysis of a Ce(NO_3_)_3_–sodium tripolyphosphate complex under different temperature regimes (60 °C (CePO_4_-1) and 90 °C (CePO_4_-2)), their physicochemical characterization (using transmission electron microscopy, X-ray diffraction, X-ray photoelectron spectroscopy, dynamic light scattering, UV spectroscopy), and the evaluation of their biocompatibility and biological activity in relation to human cells involved in skin regeneration. Their biological activity was evaluated on human dermal fibroblasts (BJ TERT) and keratinocytes (HaCaT) using MTT assays and direct cell counting across a broad concentration range (10^−2^–10^−4^ M). Synthesis temperature critically governed the atomic structure without significantly altering NPs size. CePO_4_-1 (4.7 ± 1.2 nm) was X-ray amorphous with a high density of oxygen vacancies and a mixed Ce^3+^/Ce^4+^ valence state on the surface (the physical basis for its antioxidant enzyme-mimetic and pro-regenerative activity); CePO_4_-2 (5.2 ± 0.9 nm) formed highly crystalline hexagonal rhabdophane. Both nanoparticle types demonstrated biocompatibility. A dose-dependent stimulating effect of NPs on the metabolism and proliferation of fibroblasts (by 1.14–1.45 times) was established. In contrast, keratinocytes exhibited dose-dependent metabolic suppression at higher concentrations (10^−2^–10^−3^ M), while remaining unaffected at 10^−4^ M. The amorphous, defect-rich CePO_4_-1 exhibited pronounced biological activity, significantly stimulating fibroblast metabolic (up to 128%) and proliferative (up to 145%) activity. Thus, the temperature control during the CePO_4_ NPs synthesis enables the “physical programming” of biological activity, presenting a promising strategy for the development of biocompatible, enzyme-mimetic and redox-mediated wound-healing nanodrugs.

## 1. Introduction

Chronic wounds and impaired tissue healing remain one of the critical challenges in modern medicine, necessitating the development of novel and effective therapeutic approaches [1]. In recent years, considerable research attention has been directed toward the application of nanomaterials in regenerative medicine owing to their unique physicochemical properties, high specific surface area, and capacity for active interaction with biological systems at the cellular level [1,2,3,4]. Among the various types of nanoparticles, particular interest has been directed toward nanoparticles of rare-earth metal compounds exhibiting variable valence, specifically those of cerium. The most extensively studied representative of this class is nanoceria (cerium oxide nanoparticles), the regenerative potential of which has been comprehensively documented in numerous systematic and critical reviews [5,6,7,8]. Recent studies have further elucidated that the pro-regenerative effects of nanoceria are mediated through the modulation of intracellular reactive oxygen species (ROS) levels and the activation of key signaling pathways involved in cell proliferation and migration [9,10].

From the perspective of surface physics, the presence of alternating Ce^3+^ rare-earth ions and oxygen vacancies on the nanocrystal facets, arising from surface hydration-induced disordering, generates a high density of active sites for the reversible switching of the Ce^3+^/Ce^4+^ redox state [11,12]. This endows the nanoparticles with potent enzyme-mimetic activity, enabling them to efficiently catalyze the dismutation of superoxide radicals and the decomposition of hydrogen peroxide, thereby protecting tissues and cells, including dermal fibroblasts, from oxidative stress during the inflammatory phase [13,14,15]. The therapeutic implications of this catalytic antioxidant mechanism have been substantiated by in vivo studies demonstrating accelerated wound closure and enhanced collagen deposition in animal models treated with cerium-based nanomaterials [16,17,18].

However, while the beneficial effects of cerium oxide nanoparticles have long been studied by numerous researchers from different countries, research into the potential applications of cerium phosphate nanoparticles, particularly in biomedicine, is relatively new. Currently, there is a gap in knowledge regarding CePO_4_ in wound healing. Therefore, the study of rare earth element phosphates, in particular cerium phosphate (CePO_4_), is a new and promising direction. At the same time, while nanoceria has been extensively studied for wound healing, its clinical translation is partly limited by concerns over potential pro-oxidant effects at high doses and the difficulty of tracking its biodistribution and degradation in vivo. CePO_4_ addresses several of these limitations while retaining the redox activity essential for modulating oxidative stress—one of the principal pathogenetic factors impairing normal tissue repair [19,20,21,22,23,24]. Specifically, comparative toxicological assessments have revealed that CePO_4_ nanoparticles may exhibit a more favorable safety profile than their oxide counterparts, with reduced potential for inducing adverse cellular responses and a lower propensity for uncontrolled ROS generation [24]. In addition, the relatively low solubility of CePO_4_ ensures sustained, localized redox activity at the wound site without the abrupt osmotic shifts associated with readily soluble cerium salts, thereby providing prolonged therapeutic action [20,21]. Furthermore, unlike CeO_2_, the phosphate host matrix serves as a versatile platform for functionalization and offers intrinsic luminescence capabilities [24,25,26], enabling label-free optical monitoring of material degradation directly within the wound bed. However, despite the known temperature dependence of CePO_4_ crystallization, the systematic relationship between synthesis temperature, the resulting degree of crystallinity (amorphous vs. crystalline), and enzyme-mimetic bioactivity in human skin cells has not been previously investigated, representing a critical knowledge gap that the present study aims to address. These combined properties—enhanced biocompatibility, controlled dissolution kinetics, and optical traceability—position CePO_4_ as a distinct and advantageous alternative to CeO_2_ for regenerative wound care.

Cerium orthophosphates, particularly in the form of nanohydrated rhabdophane (CePO_4_·nH_2_O), have attracted attention in regenerative medicine also owing to their relatively low solubility [20,21,22,27,28], which ensures a prolonged action of the material without abrupt changes in osmotic pressure within the wound, in contrast to readily soluble salts. This effect is manifested through the oxidative passivation of the cerium orthophosphate surface, leading to the formation of an insoluble Ce(IV) hydroxide–phosphate film. The kinetics of dissolution and transformation of rare-earth phosphate nanoparticles in biological media have been systematically investigated, providing a mechanistic basis for their sustained bioactivity [29].

It is also important to note that the CePO_4_ crystal lattice exhibits a low maximum phonon energy (vibrational frequency of approximately 1000–1100 cm^−1^), as established by Raman and FTIR spectroscopy [22,30,31]. Such a low phonon energy of the host matrix effectively suppresses non-radiative relaxation pathways of excited states of lanthanide activator ions, for instance, Tb^3+^ or Eu^3+^ [32], thereby providing a direct route toward the fabrication of biocompatible luminescent scaffolds. These composite dressings would enable the optical monitoring of material biodegradation directly within the wound bed without the use of additional organic dyes, relying exclusively on the photoluminescence of the doped phosphate matrix. The potential of lanthanide-doped nanophosphors for multimodal imaging and biosensing has been extensively explored, highlighting their utility in theranostic wound care applications [21,33,34].

Based on the foregoing, it can be hypothesized that nanosized cerium phosphate may combine the beneficial properties of both oxides and phosphates, which could be harnessed for the development of novel highly effective regenerative materials. However, at the initial stages, it is expedient to establish a suitable basis for the synthesis method of cerium phosphate nanoparticles by investigating their properties, stability, and reproducibility.

The aim of the present work is the synthesis of hydrated cerium phosphate nanoparticles via controlled hydrolysis under different temperature regimes, their comprehensive comparative physicochemical characterization, and the evaluation of their biological activity with respect to wound-healing processes in human cells involved in skin tissue regeneration.

The selection of the synthesis method for CePO_4_·nH_2_O nanoparticles intended for wound-healing applications in this study was dictated by the necessity of obtaining readily dispersible colloidal systems in aqueous media with controlled size and crystal structure. As a basis, we adopted the approach proposed by V.Buissette et al. [35], which involves the hydrolysis of a mixture of lanthanide salts and sodium tripolyphosphate (TPP) in an aqueous medium. This method offers several significant advantages: it proceeds under mild conditions (aqueous solution, moderate temperatures), does not require the use of toxic organic solvents, and enables the production of nanoparticles with a narrow size distribution owing to the dual function of polyphosphate groups—acting both as a source of orthophosphate ions for crystallization and as a stabilizing agent preventing aggregation. Furthermore, it was demonstrated in the original study that the nanoparticle surface can be additionally modified with polyphosphates, thereby opening possibilities for subsequent functionalization with biologically active molecules.

## 2. Materials and Methods

### 2.1. Synthesis of Cerium Phosphate Nanoparticles

Nanoparticles of hydrated cerium orthophosphate were synthesized by the controlled hydrolysis of the complex formed upon the reaction of Ce(NO_3_)_3_ and Na_5_P_3_O_10_ (sodium tripolyphosphate, TPP) in aqueous solution [35]. The methodology employed herein aligns with established green synthesis principles for lanthanide phosphate nanomaterials, which prioritize aqueous media and moderate conditions to ensure biocompatibility of the final product [36]. Fifty milliliters of a 0.1 M solution of cerium(III) nitrate hexahydrate (99.995% in terms of rare-earth elements, LANHIT LLC, Moscow, Russia) was mixed with 50 mL of a 0.1 M solution of sodium tripolyphosphate (≥98%, Sigma-Aldrich, St. Louis, MI, USA). The resulting clear solution was maintained for one hour at a temperature of 60 °C (CePO_4_-1) or 90 °C (CePO_4_-2), during which turbidity developed. After one hour, the reaction mixture was cooled and transferred into a dialysis bag (44 mm, 3.5 kDa, M-Cel, Lombard, IL, USA), and dialysis was performed against 1 L of distilled water for 5 days, with daily water changes. After dialysis, the dispersions were filtered through 0.22 µm sterile PVDF filters. The resulting transparent dispersions contained CePO_4_·xH_2_O nanoparticles at a concentration of 7 mg/mL (CePO_4_-1, synthesis at 60 °C) or 12 mg/mL (CePO_4_-2, synthesis at 90 °C).

In accordance with the recommendations for improving stability, 0.1% sodium hexametaphosphate stabilizer was added to the final sol [35]. In attempted syntheses without the use of Na_6_P_6_O_18_, the nanoparticles precipitated within 30 days, which hindered their characterization and further use in biomedicine.

The concentration of the obtained sols was determined gravimetrically by carefully evaporating a defined volume of the dispersion in a drying oven at 50 °C to constant weight. The mass was determined using an analytical balance A&D GR-120 (A&D Company, Tokyo, Japan).

To prepare sols of the required concentration for biomedical investigation methods (0.00001–0.01 M), the initial sols of 7 mg/mL (CePO_4_-1, synthesis at 60 °C) and 12 mg/mL (CePO_4_-2, synthesis at 90 °C) were diluted with sterile bidistilled water. Taking into account M_m_(CePO_4_) = 235.09 g/mol, the initial concentration of CePO_4_-1 was 0.03 M, and that of CePO_4_-2 was 0.05 M. Accordingly, to prepare the highest concentration (10^−2^ M), CePO_4_-1 was diluted 3-fold and CePO_4_-2 was diluted 5-fold.

The present study reports the results obtained for stable sols stored at room temperature for 2–3 months, after which investigations on human cell lines were performed.

### 2.2. Study Design

For the subsequent physicochemical and biomedical characterization, the obtained sols of samples CePO_4_-1 and CePO_4_-2 were used, as well as nanocrystalline cerium phosphate powders obtained by drying the dispersions at 50 °C.

The following physicochemical characterization methods were employed: transmission electron microscopy (TEM), X-ray phase analysis (XRPA), X-ray photoelectron spectroscopy (XPS), dynamic light scattering (DLS) with determination of the hydrodynamic radius and zeta potential, and UV spectroscopy.

Biocompatibility and biological activity were assessed on living cells involved in skin regeneration and wound healing (human fibroblasts and keratinocytes) using direct cell counting and the MTT assay after co-culture with the nanosols over a broad range of CePO_4_ concentrations (0.00001–0.01 M). The concentrations were obtained by the addition of sterile bidistilled water.

### 2.3. Physicochemical Characterization Methods

#### 2.3.1. Transmission Electron Microscopy

The morphology and average size of the nanoparticles were determined by transmission electron microscopy (TEM) using a JEOL JEM-2100 microscope (JEOL, Akishima, Tokyo, Japan) at an accelerating voltage of 200 kV at the Collective Use Center “Materials Science and Metallurgy” of the National University of Science & Technology MISIS. Dispersions of CePO_4_·nH_2_O nanoparticles were drop-cast onto a carbon grid and dried. The average nanoparticle size was evaluated by measuring at least 100 particles along their longest dimension. Statistical processing was performed using Origin software (version b9.2.196).

#### 2.3.2. UV Spectroscopy

Absorption spectra of the cerium phosphate dispersions in the wavelength range of 300–800 nm were recorded using a Varian Cary 50 spectrophotometer (Agilent Technologies, Santa Clara, CA, USA).

#### 2.3.3. X-Ray Phase Analysis and X-Ray Photoelectron Spectroscopy

For the investigation by XRD and XPS, the synthesized cerium phosphate dispersions were carefully evaporated at 50 °C in a drying oven until constant weight was achieved.

The phase composition and crystal structure of the synthesized cerium phosphate nanoparticles were studied by powder X-ray diffraction (XRD) using a DRON-4-07 diffractometer (LNPO “Burevestnik”, Saint Petersburg, Russia). X-ray diffraction patterns were recorded using a DRON-4-07 diffractometer with Cu Kα radiation (λ = 1.5418 Å) at an accelerating voltage of 40 kV and a tube current of 20 mA. The measurements were performed at diffraction angles 2Θ from 10° to 120° with a step of 0.1°. Qualitative phase analysis was carried out by comparing the diffraction patterns with the PDF2 database. The average crystallite size D was calculated using the Scherrer equation:

(1) $$D=\frac{k\lambda }{cos\theta \sqrt{\beta_{exp}^{2}-\beta_{app}^{2}}}$$

where λ is the wavelength of X-ray radiation (nm); β_exp_ is the observed peak width at half maximum and β_app_ is the instrumental broadening (rad); θ is the diffraction angle; and k is a coefficient equal to 0.9.

X-ray photoelectron spectroscopy (XPS) measurements were obtained using an OMICRON ESCA+ spectrometer (Scienta Omicron, Uppsala, Sweden) with an Al Kα X-ray source (E = 1486.6 eV, 252 W). To compensate for local charging of the analyzed surface, a CN-10 charge neutralizer was used with an emission current of 6 μA and a beam energy of 1 eV. The analyzer pass energy was 20 eV. The main state of the C 1s core level was used as a reference with a binding energy (BE) of 285 eV.

#### 2.3.4. Dynamic Light Scattering

The average hydrodynamic size and zeta potential of the cerium phosphate nanoparticles were determined by the dynamic light scattering (DLS) method using a Malvern Zetasizer Nano ZS (Malvern Panalytical, Worcestershire, UK). Prior to analysis, the dispersions were filtered through a 0.22 µm PVDF filter.

### 2.4. Biological Investigation Methods on Cell Lines

Biocompatibility, cytotoxicity, and the effects of nanosols at concentrations of 10^−2^ M, 10^−3^ M, and 10^−4^ M on metabolic and proliferative activity were evaluated using the MTT assay and direct cell counting with determination of the dead/live cell ratio after 72 h of co-culture with the nanocomposites, according to proven and standardized methods described in detail previously [20,37]. The selection of these specific cell lines was guided by the established roles of dermal fibroblasts and epidermal keratinocytes as key cellular mediators in the wound healing cascade, as reviewed in detail elsewhere [38,39].

In the biomedical study, concentration (10^−2^ M, 10^−3^ M, 10^−4^ M) means the concentration of the initial nanocomposite sol added to the well in a volume of 10%. Distilled water added to the wells in the same volume as the nanocomposites (10% *v*/*v*) served as the control.

The MTT assay was performed with 12 replicates (n = 12); cell counts were determined in 6 wells of each CePO_4_ subgroup (n = 6).

#### 2.4.1. Cell Lines and Their Cultivation

The experiments were performed on the HaCaT and BJ TERT cell lines. The BJ TERT cell line is a modified line of primary human fibroblasts obtained by introducing a specific genetic element—the human catalytic subunit of the telomerase enzyme. The line originated from the American Type Culture Collection (ATCC) (Manassas, VA, USA). The cells are fibroblast-like and exhibit an adhesive growth pattern.

The HaCaT cell line consists of spontaneously immortalized non-cancerous human keratinocytes capable of unlimited division while exhibiting normal differentiation. The cells were obtained from the cell bank of the N.N. Blokhin National Medical Research Center of Oncology, Ministry of Health of the Russian Federation (Moscow, Russia) and are characterized by an adhesive growth pattern.

Cell culture was performed in Petri dishes (SPL Life Sciences, Pocheon-si, Republic of Korea) using a standard protocol in complete DMEM medium with high glucose (PanEco, Moscow, Russia). Cell cultures were maintained in a CO_2_ incubator (Binder, Tuttlingen, Germany) under standard controlled conditions (5% CO_2_, 37 °C) and controlled humidity.

#### 2.4.2. MTT Assay

To assess cell proliferative activity, an MTT assay was performed. Briefly, after incubation, the culture medium was aspirated, MTT reagent (PanEco, Moscow, Russia) was added, and the sample was incubated in an incubator at a constant temperature of 37 °C. The MTT working solution was then removed, and dimethyl sulfoxide (neoFroxx, Einhausen, Germany) was added to the wells. The plate was incubated at room temperature for 5 min until the formazan was completely dissolved and the solution was uniform across the wells. Optical density was then measured using a Multiscan Labsystems spectrophotometer (Finland) at a wavelength of 540 nm. Results were expressed as relative optical density (OD).

#### 2.4.3. Determination of Proliferative Activity and the Dead/Live Cell Ratio Using Trypan Blue Staining

Cell counting and determination of the ratio of live to dead cells were performed automatically using a Countess II Automated Cell Counter (ThermoScientific, Waltham, MA, USA). The culture medium in which the cells had been cultured was aspirated from the well. Cells were then detached using a Versene/trypsin solution (PanEco, Gorki Leninskiye, Russia). Detached cells were transferred to the previously collected samples from the corresponding wells. The resulting samples were carefully pipetted, after which 0.4% trypan blue solution (Servicebio, Wuhan, China) was added, and direct cell counting was performed using the appropriate slides. This determined the total cell concentration per unit volume and the percentage of live versus dead cells.

### 2.5. Statistical Processing

The statistical program SPSS 25.0 (IBM, Armonk, NY, USA) was used to process the results of the biomedical investigations. The Shapiro–Wilk and Kolmogorov–Smirnov criteria were used to evaluate the normality of indicator distributions. In the cell experiments, the mean value was determined in the control group. Relative to the mean value in each experiment, the percentages in the studied groups were calculated to obtain the final figure expressed as the percentage of the control. For the comparative analysis of multiple subgroups, one-way ANOVA was performed using post hoc comparisons and Dunnett’s test (for comparison with controls). Independent samples that did not follow a normal distribution were compared using the Kruskal–Wallis test. If the Kruskal–Wallis test revealed a statistically significant difference among all groups, pairwise comparisons were performed using the Mann–Whitney U test with Bonferroni correction. For all criteria and tests, differences were considered statistically significant at *p* < 0.05.

## 3. Results

### 3.1. Transmission Electron Microscopy Results

#### TEM of Cerium Phosphate Samples

**Figure 1:**
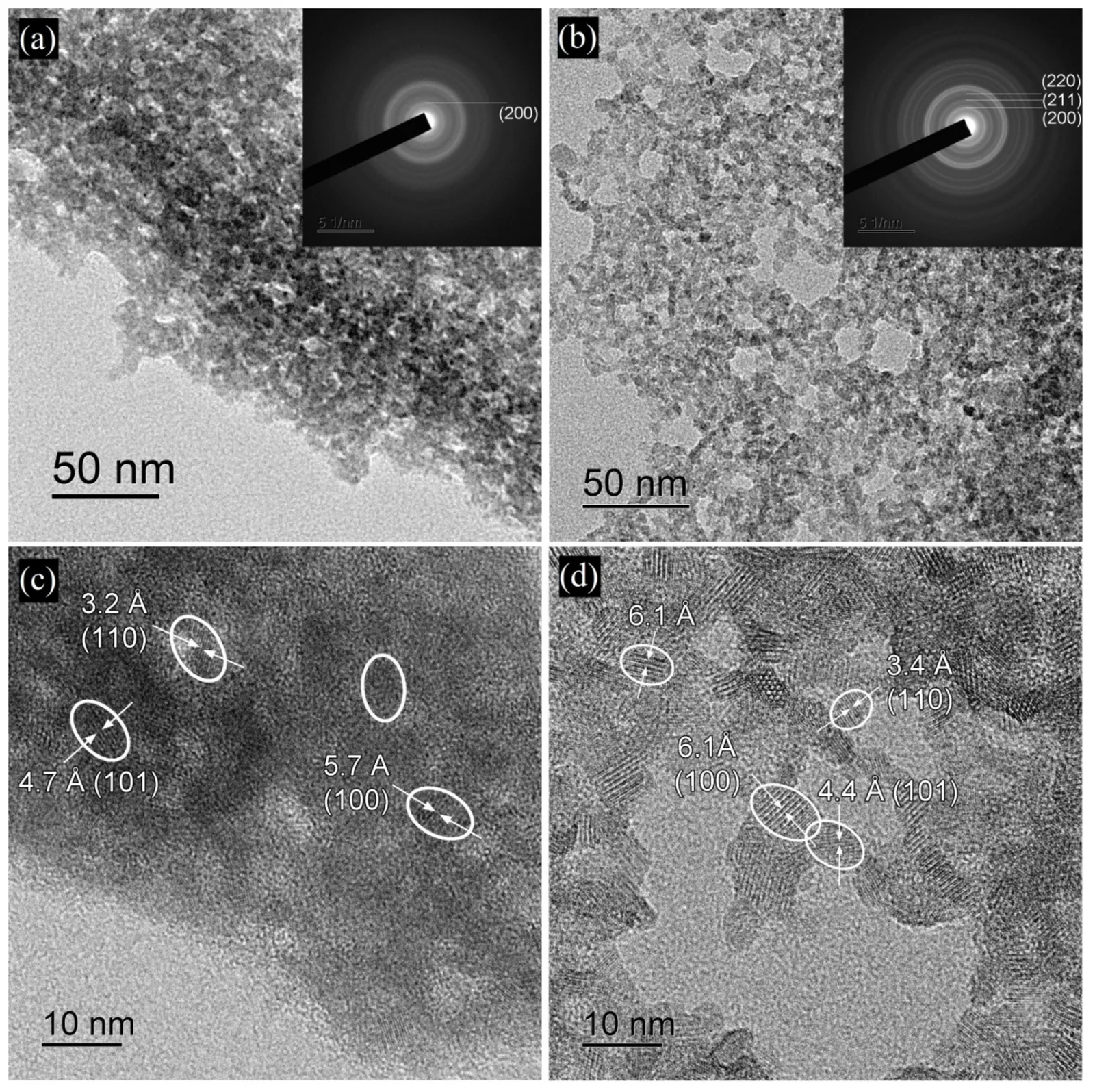
TEM images at different magnifications for samples CePO_4_-1 (**a**,**c**) and CePO_4_-2 (**b**,**d**). Insets in (**a**) and (**b**) show electron diffraction patterns from the corresponding sample area.

**Figure 2:**
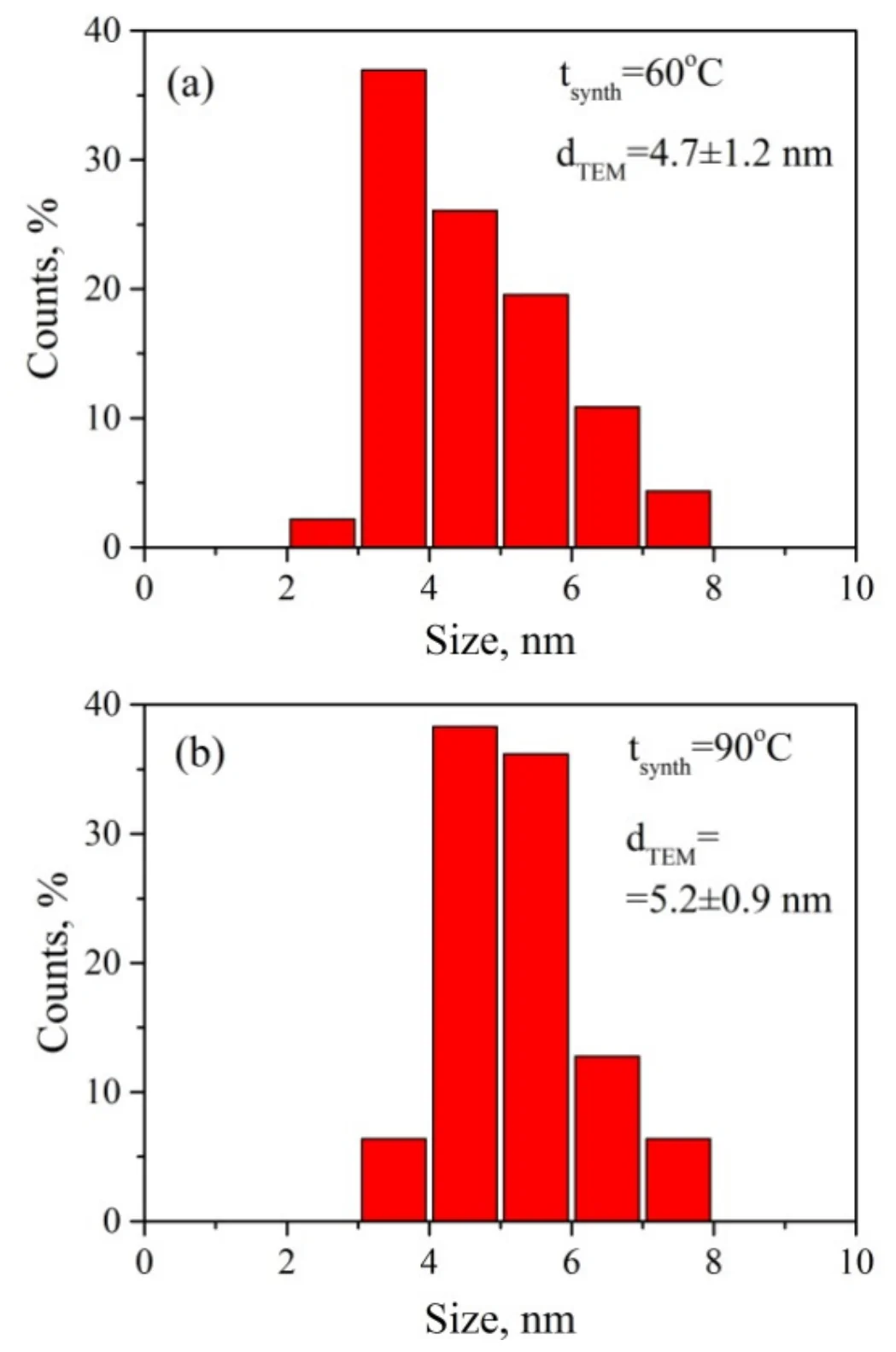
Nanoparticle size distribution histograms for samples CePO_4_-1 (**a**) and CePO_4_-2 (**b**).

TEM images of the CePO_4_-1 sample synthesized at 60 °C reveal strongly agglomerated, ultrafine nanoparticles with an average size of 4.7 ± 1.2 nm (Figure 1a,c and Figure 2a). The particles are approximately spherical to ellipsoidal, although individual particle boundaries are frequently obscured by aggregation. Increasing the synthesis temperature to 90 °C results in only a modest increase in the average particle size, to 5.2 ± 0.9 nm (Figure 2b), while producing a pronounced increase in crystallinity (Figure 1b,d). The CePO_4_-2 nanoparticles retain an approximately spherical-to-ellipsoidal morphology, with some particles exhibiting weak elongation.

The selected-area electron diffraction (SAED) patterns (insets in Figure 1a,b) are consistent with the X-ray diffraction results and demonstrate a substantial difference in structural ordering between the two samples. The CePO_4_-1 sample exhibits diffuse, strongly broadened diffraction rings superimposed on a pronounced diffuse background, indicating very small coherent crystalline domains and substantial structural disorder. In contrast, the SAED pattern of CePO_4_-2 contains a series of considerably sharper and well-resolved Debye–Scherrer rings characteristic of polycrystalline rhabdophane CePO_4_·nH_2_O, confirming the substantially higher degree of crystallographic ordering attained at 90 °C.

High-resolution TEM observations further support this interpretation. In CePO_4_-1, lattice fringes are observed only locally within the predominantly disordered nanoparticle aggregates, demonstrating the presence of small crystalline domains. Measured interplanar spacings of approximately 4.7, 3.2, and 2.8 Å are within the range of low-index lattice spacings expected for rhabdophane-type CePO_4_·nH_2_O. The value of approximately 2.8 Å is consistent with the (102) reflection of the conventional rhabdophane structure.

In comparison, CePO_4_-2 exhibits extended and clearly resolved lattice fringes throughout a substantially larger fraction of the nanoparticles, confirming its higher crystallinity. Interplanar spacings of approximately 6.05 and 4.40 Å agree closely with the (100) and (101) lattice spacings, respectively, of the conventional rhabdophane CePO_4_·nH_2_O structure (PDF 35-0614). Overall, the TEM, HRTEM, and SAED results indicate that increasing the synthesis temperature from 60 °C to 90 °C has only a minor effect on the mean nanoparticle size but markedly promotes crystallographic ordering and growth of coherent rhabdophane domains.

Thus, transmission electron microscopy data demonstrate that varying the temperature within the range of 60–90 °C enables the controlled synthesis of cerium phosphate nanoparticles with identical morphology and size but with fundamentally different internal structures, ranging from amorphous (with elements of short-range order) to fully crystalline with the rhabdophane structure. This provides a basis for the subsequent rigorous comparison of the influence of the degree of crystallinity on biological activity.

### 3.2. UV Spectroscopy Results

**Figure 3:**
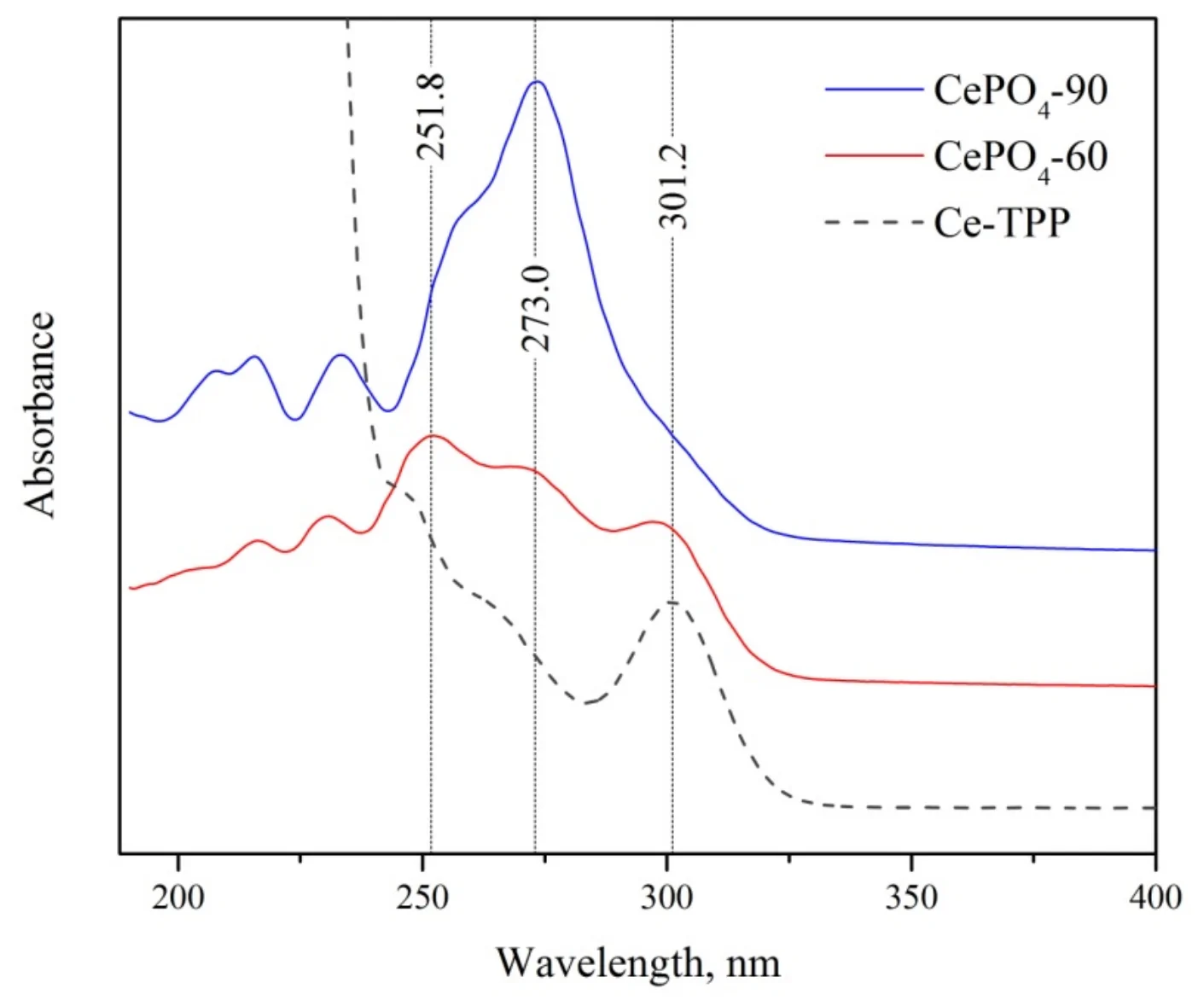
Optical absorption spectra: cerium nitrate–sodium tripolyphosphate complex (dashed line), dispersion of sample CePO_4_-1 after dialysis (red line), and dispersion of sample CePO_4_-2 after dialysis (blue line).

Dispersions of CePO_4_·nH_2_O nanoparticles in water exhibit light absorption solely in the UV range, with nanoparticles synthesized at different temperatures also displaying differences in their absorption spectra (Figure 3). The initial Ce(NO_3_)_3_–TPP complex is characterized by an absorption peak at 301 nm, which gradually disappears during its hydrolysis and the formation of CePO_4_·nH_2_O nanoparticles. The spectrum of the CePO_4_-1 sample contains a weak peak at 301 nm and a series of new peaks at 273 nm and 252 nm, characteristic of the amorphous CePO_4_·nH_2_O phase. In the case of the crystalline CePO_4_·nH_2_O phase (CePO_4_-2), the peak at 301 nm virtually disappears, and a strong absorption peak emerges at 273 nm with a shoulder at 256 nm. The absorption peak at 273 nm, observed for both samples, can presumably be considered characteristic of CePO_4_·nH_2_O nanoparticles of the rhabdophane phase. The spectra also contain a series of absorption peaks in the shorter-wavelength region (<240 nm), which can only be observed in dialyzed dispersions.

Thus, UV spectroscopy demonstrates that the synthesis temperature influences the chemical kinetics: 90 °C is required for the complete decomposition of the precursor and the formation of a well-defined crystalline structure with a pronounced maximum at 273 nm, whereas 60 °C leads to an intermediate state (an amorphous matrix with rhabdophane nuclei and residual complexes on the surface). Notably, purification by dialysis is an essential step for the correct registration of the complete spectral characteristics of the nanoparticles themselves in the short-wavelength UV region.

The structured band in the 240–310 nm region of rhabdophane dispersions is predominantly attributed to the parity-allowed Ce^3+^ 4f1 → 4f05d1 transitions. The partially resolved features at approximately 252, 273, and 301 nm can be associated with crystal-field splitting of the Ce^3+^ 5d states, whose energies are sensitive to the local coordination environment. The CePO_4_-2 sample shows a pronounced band near 273 nm, whereas the corresponding features of CePO_4_-1 are broader, consistent with the higher crystallographic ordering of the former observed by XRD, SAED, and HRTEM. In the less crystalline CePO_4_-1 sample, structural disorder, hydration, and a higher proportion of heterogeneous surface Ce sites likely cause stronger inhomogeneous broadening of the 4f → 5d absorption bands.

### 3.3. Dynamic Light Scattering: Determination of Zeta Potential and Hydrodynamic Diameter of Nanoparticles in Dispersions

**Figure 4:**
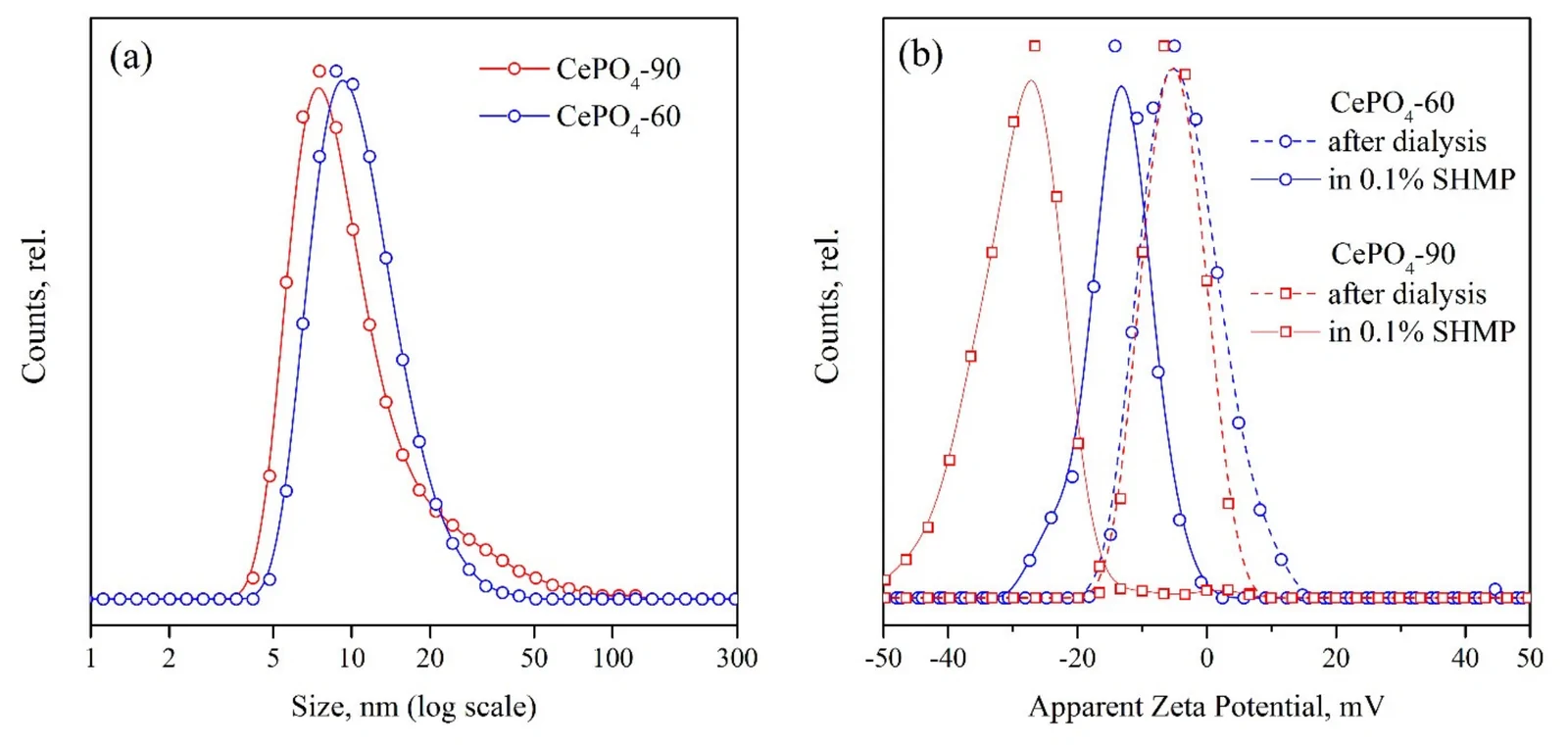
Hydrodynamic diameter of nanoparticles in aqueous dispersions of samples CePO_4_-1 and CePO_4_-2 (**a**); zeta potential of CePO_4_-2 nanoparticles after dialysis and in 0.1% sodium hexametaphosphate (SHMP) solution (**b**).

Evaluation of the average hydrodynamic diameter of CePO_4_·nH_2_O nanoparticles in aqueous dispersions yields values in the range of 7–10 nm, indicating a substantial thickness of the hydration shell (Figure 4a). Immediately after dialysis, the nanoparticles exhibit a slightly negative zeta potential (approximately −3 mV), which accounts for their low colloidal stability. The stability of CePO_4_·nH_2_O nanoparticle dispersions can be significantly enhanced by employing sodium hexametaphosphate as a stabilizer, leading to an increase in zeta potential to −13 mV in a 0.1% solution for CePO_4_-1 and −30 mV for CePO_4_-2 (Figure 4b). Long-term stability of the dispersions can also be ensured by reducing their concentration to 1 mg/mL and storing them at a reduced temperature (5–10 °C).

The surface of rhabdophane nanoparticles is strongly hydrophilic and can interact with water through surface phosphate and hydroxylated Ce sites. Consequently, the hydrodynamic diameter includes contributions from the nanoparticle, its interfacial hydration environment and associated counterions, as well as possible small particle aggregates. The larger hydrodynamic diameter compared with the primary particle sizes observed by HRTEM is consistent with this interpretation. Interestingly, despite its slightly larger primary-particle size measured by TEM, the more crystalline CePO_4_-2 sample exhibits a smaller characteristic hydrodynamic size than CePO_4_-1. This may reflect differences in surface structural disorder, hydration, and/or loose particle association.

### 3.4. X-Ray Diffraction Analysis Results

**Figure 5:**
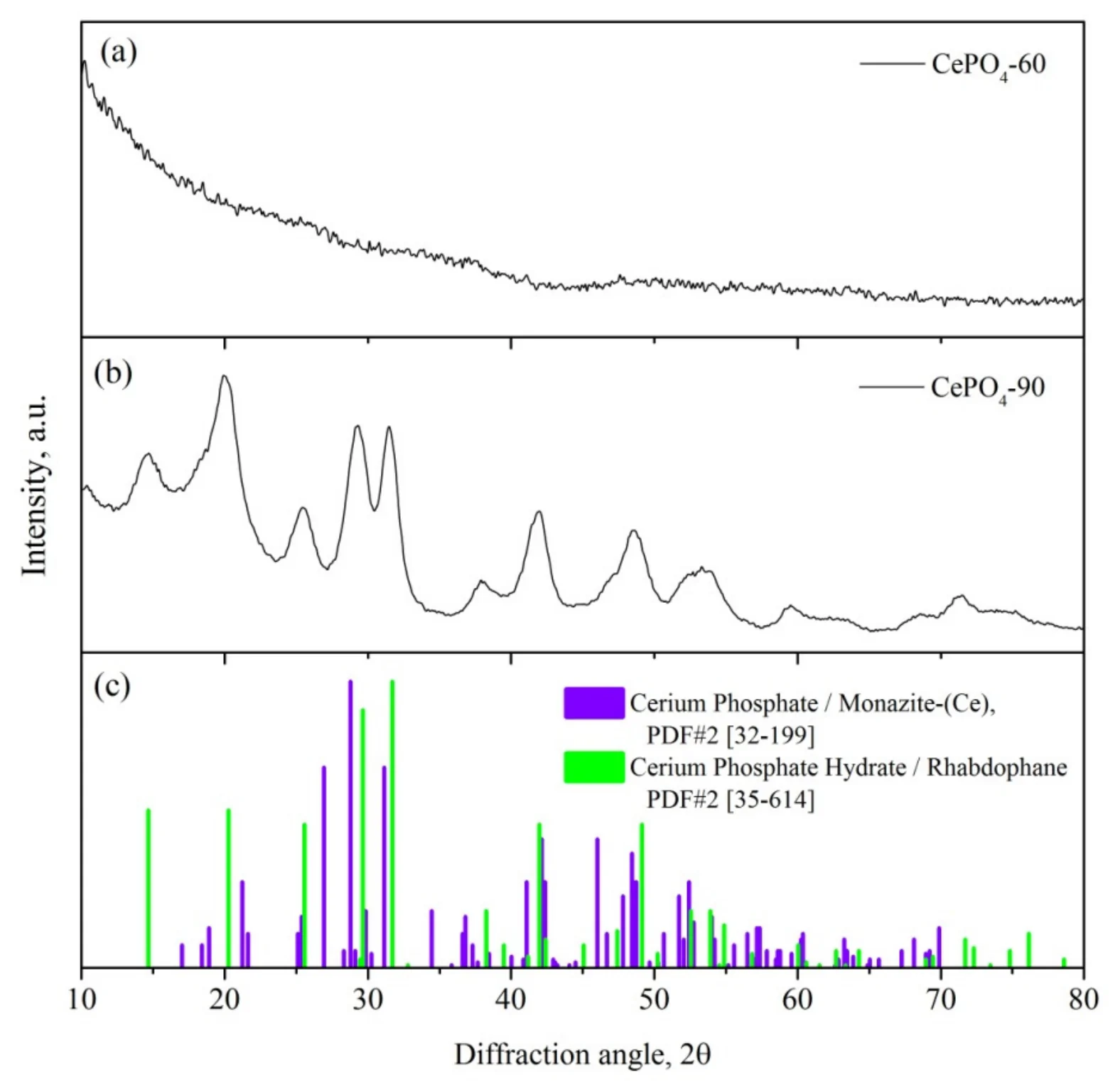
Diffraction patterns of samples CePO_4_-1 (**a**), CePO_4_-2 (**b**), and standard stick patterns of the monazite (CePO_4_, PDF#2 32-199) and rhabdophane (CePO_4_·nH_2_O, PDF#2 35-614) phases (**c**).

Powder X-ray diffraction patterns of the synthesized cerium phosphate nanoparticles are presented in Figure 5. Sample CePO_4_-1 is X-ray amorphous; no reflections are detected in its diffraction pattern (Figure 5a). Sample CePO_4_-2 exhibits significantly higher crystallinity, displaying a series of broadened peaks in its diffraction pattern (Figure 5b) attributable to the hexagonal rhabdophane phase (CePO_4_·nH_2_O). The sample is single-phase, i.e., peaks corresponding to other crystalline phases are absent. Estimation of the average crystallite size of sample CePO_4_-2 using the Scherrer equation yields values in the range of 6.5–7.9 nm. The refined parameters of the hexagonal unit cell of sample CePO_4_-2 were determined to be a = b = 7.01 Å, c = 6.43 Å.

X-ray phase analysis fully corroborates the structural transitions revealed by TEM and UV spectroscopy. Increasing the synthesis temperature to 90 °C is critically important for the formation of a well-crystallized, single-phase rhabdophane structure with a crystallite size of approximately 7 nm and lattice parameters close to the reference values. At 60 °C, an X-ray amorphous product is formed, indicating insufficient thermal energy to complete the crystallization reactions and fully decompose the initial complexes. The temperature-dependent phase evolution observed here is consistent with the general crystallization behavior of rare-earth phosphates, wherein the dehydration and structural ordering of the rhabdophane phase are kinetically controlled processes [40,41].

Thus, the deliberate variation in the synthesis temperature enables the production of both amorphous and crystalline cerium phosphate nanoparticles with virtually identical morphology, thereby providing a foundation for the rigorous comparison of the influence of the degree of crystallinity on their physicochemical and biological properties.

### 3.5. XPS Results

**Figure 6:**
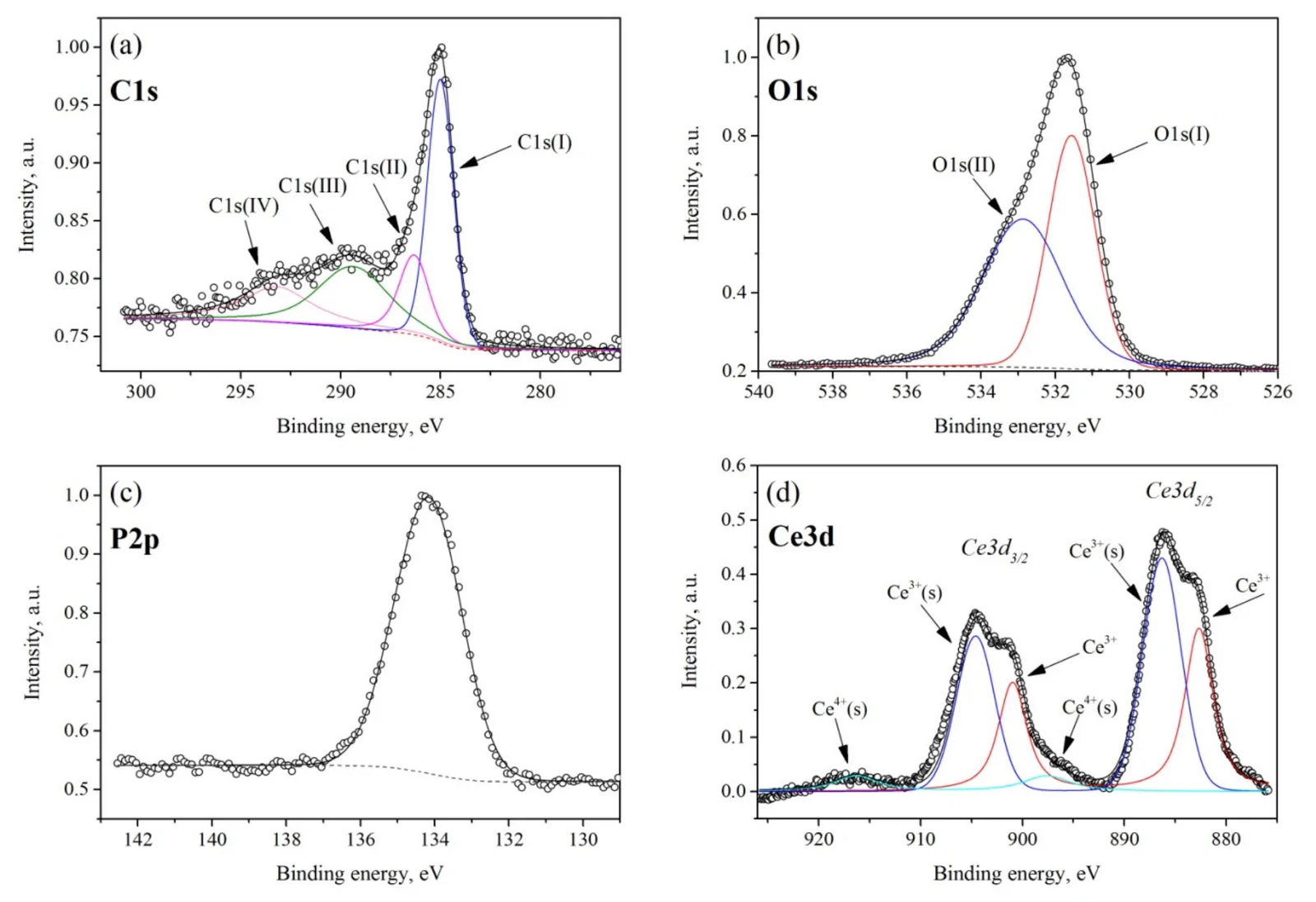
X-ray photoelectron spectra of sample CePO_4_-1 in the regions: C 1s (**a**), O 1s (**b**), P 2p (**c**), and Ce 3d (**d**).

X-ray photoelectron spectra of sample CePO_4_-1 in the C 1s, O 1s, P 2p, and Ce 3d regions are shown in Figure 6. The oxygen spectrum is represented by two states, with relative intensities of 47% and 53% for O 1s (I) and O 1s (II), respectively. The charge state with a binding energy of 531.6 eV (O 1s (I)) can be assigned to the “lattice” oxygen of the sample, whereas the state with a binding energy of 532.9 eV may correspond to surface oxygen atoms bound to hydroxyl groups. Phosphorus in the sample exhibits a single charge state with a binding energy of 133.9 eV (for P 2p_3_/_2_), characteristic of the 5+ oxidation state of phosphorus in metal phosphates. The cerium spectrum has a complex appearance due to the emergence of characteristic satellites in addition to the Ce 3d_5_/_2_ (882.6 eV) and Ce 3d_3_/_2_ (900.9 eV) peaks, which are separated from the main peaks by 3.6 eV. According to the literature data, such a spectral profile is characteristic of compounds containing cerium in the +3 oxidation state [42]. On the other hand, additional low-intensity satellites at 897.6 and 916.3 eV are observed in the spectrum of sample CePO_4_-1, which are characteristic of cerium in the +4 oxidation state [43], potentially indicating partial oxidation of surface cerium atoms in the sample to Ce^4+^. Since XPS predominantly probes the near-surface region, the Ce^4+^ contribution is related to partial oxidation of under-coordinated Ce^3+^ sites at the nanoparticle surface rather than to mixed Ce^3+^/Ce^4+^ valence throughout the rhabdophane lattice. Such oxidized surface sites can appear during synthesis and subsequent exposure to water and air. Their low concentration and surface-localized character are consistent with the absence of a separate crystalline phase in the XRD and electron-diffraction data.

Calculation of the quantitative composition of the samples from the obtained photoelectron spectra yields atomic ratios of Ce:P:O = 1:1.12:4.36, which also indicates a slight excess of oxygen compared to the stoichiometric composition of CePO_4_. This excess may be attributed to the presence of water molecules in the crystal structure of rhabdophane CePO_4_·nH_2_O. The co-existence of Ce^3+^ and Ce^4+^ species on the surface of cerium-based nanoparticles is a well-documented phenomenon that directly governs their catalytic antioxidant properties, as both oxidation states are required for the reversible redox cycles underlying superoxide dismutase and catalase mimetic activities [44,45].

### 3.6. Results of Cell Line Investigations

#### 3.6.1. Results of Investigations on the Effect of CePO_4_ Sols on the Cytotoxicity and Metabolic and Proliferative Activity of Human Fibroblasts

**Figure 7:**
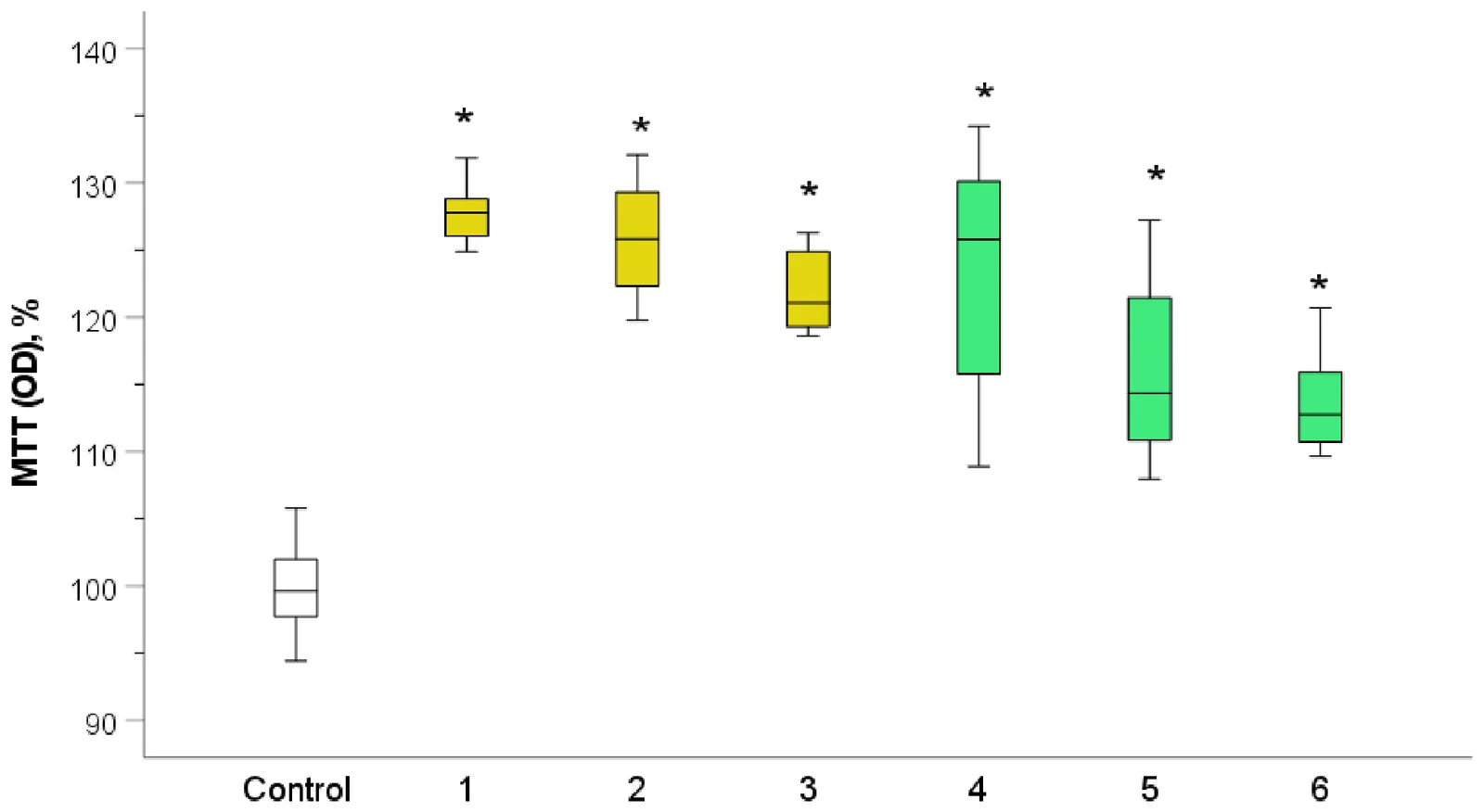
Effect of sols with different concentrations of cerium phosphate nanoparticles synthesized under different temperature regimes on the metabolic activity of human fibroblasts in the MTT assay. 1—CePO_4_-1 at 10^−2^ M, 2—CePO_4_-1 10^−3^ M, 3—CePO_4_-1 10^−4^ M, 4—CePO_4_-2 at 10^−2^ M, 5—CePO_4_-2 10^−3^ M, 6—CePO_4_-2 10^−4^ M. The Kruskal–Wallis test revealed a statistically significant difference (H = 78.4, df = 6, *p* < 0.001); *—difference from Control at *p* < 0.001.

The study established that all variants of the synthesized cerium phosphates at all concentrations significantly activated the metabolic activity of fibroblasts in the MTT assay, on average by a factor of 1.14–1.28 relative to the control (*p* < 0.01) (Figure 7). Maximum stimulation was recorded upon the addition of concentrated 10^−2^ M sols to the medium (127.7% relative to control for CePO_4_-1 at 10^−2^ M and 123.4% relative to control for CePO_4_-2 at 10^−2^ M; no differences between groups, *p* > 0.05).

At the same time, the stimulation of fibroblast metabolism was least pronounced upon co-culture with crystallized CePO_4_-2, where the average increase in OD amounted to 114–123% relative to the control. Amorphous CePO_4_-1, in turn, increased metabolic activity to 122–128% relative to the control (*p* < 0.01). However, this was at the level of a trend, and no statistically significant differences between the groups at identical concentrations were registered (*p* > 0.05).

**Figure 8:**
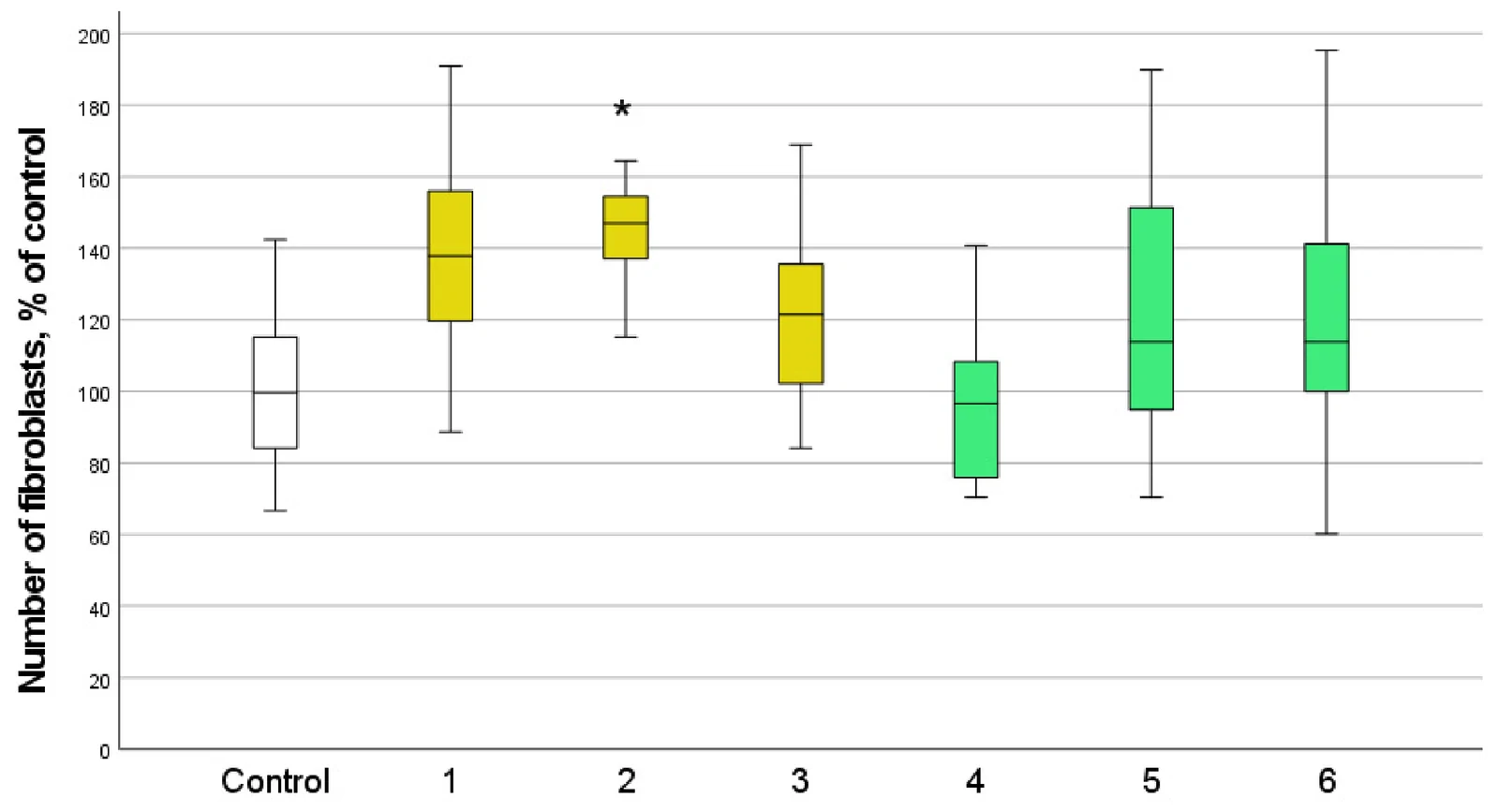
Effect of the cerium phosphate nanoparticle synthesis methodology and concentration on the proliferative activity of fibroblasts (BJTERT cell line) as determined by direct cell counting using an automated cell counter. Mean values are presented as a percentage of the Control. 1—CePO_4_-1 at 10^−2^ M, 2—CePO_4_-1 10^−3^ M, 3—CePO_4_-1 10^−4^ M, 4—CePO_4_-2 at 10^−2^ M, 5—CePO_4_-2 10^−3^ M, 6—CePO_4_-2 10^−4^ M. The Kruskal–Wallis test revealed a statistically significant difference (H = 18.7, df = 6, *p* = 0.005); *—difference from Control at *p* < 0.001.

A stimulatory effect of nanoceria on the proliferative activity of fibroblast-lineage cells was established, which was most pronounced for CePO_4_-1 (the number of fibroblasts after 72 h amounted to 121.8% relative to the control upon the addition of 10^−4^ M sol to the medium, 144.9% at 10^−3^ M, and 138.8% at 10^−2^ M). Pairwise comparisons indicated significant differences between Control and CePO_4_-1 10^−3^ M (*p* = 0.009). The other samples did not show a statistically significant difference from the control in terms of fibroblast count. CePO_4_-2 sols at concentrations of 10^−2^ and 10^−3^ M non-significantly stimulated proliferation, on average to 115–118% relative to the control (*p* > 0.05) (Figure 8). There were also no differences in this parameter between the nanosols of different groups at identical concentrations, indicating the biological equivalence of the nanoparticles.

**Figure 9:**
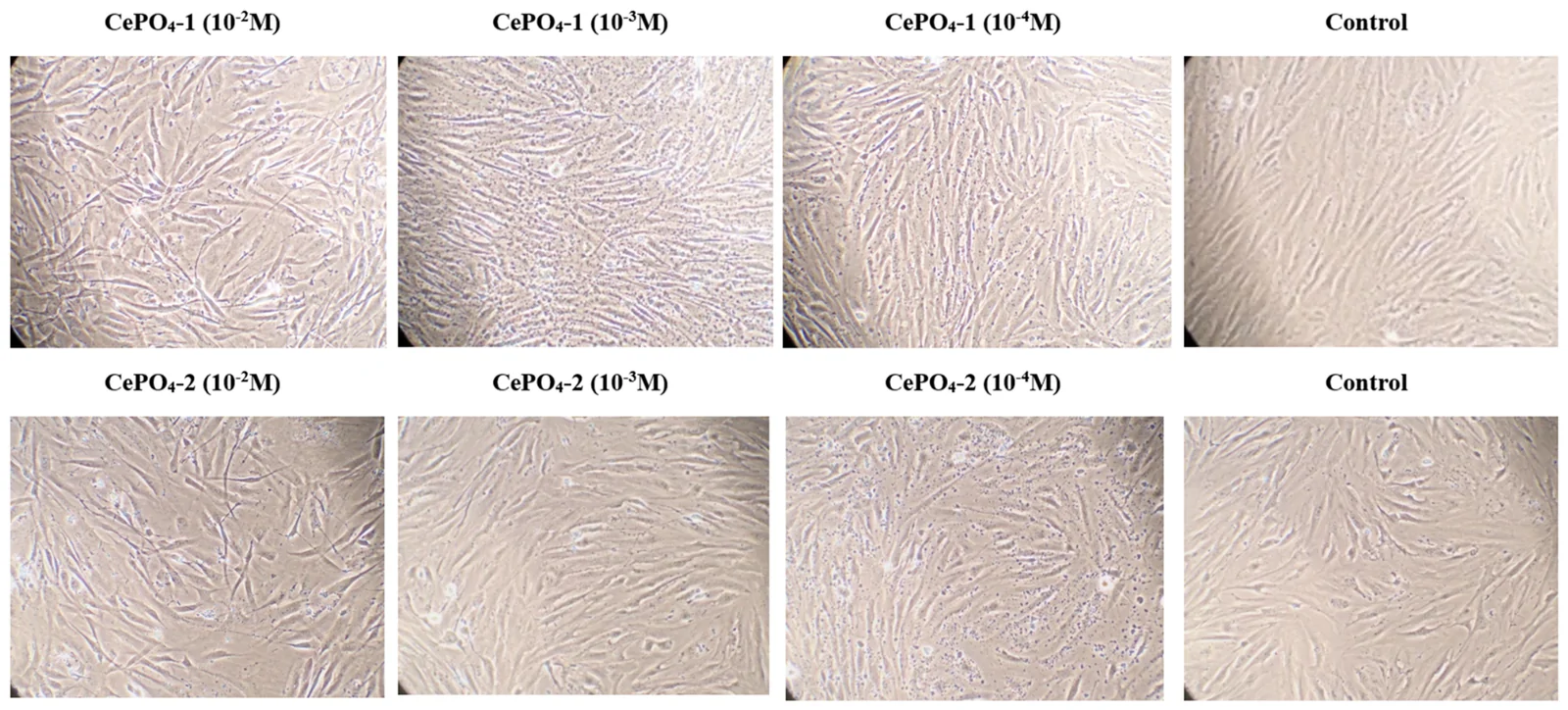
Photographs of fibroblasts after 72 h of co-cultivation with CePO_4_ of different synthesis modifications and at different concentrations.

It is important to note that no negative cytotoxic effect was registered for any of the sol variants, which may attest to their non-toxicity and cytocompatibility with respect to human fibroblasts. Analysis of the percentage of dead cells upon co-culture with all cerium phosphate samples at different concentrations revealed no cytotoxicity. The proportion of dead cells was below the detection limit of the Countess II automated cell counter, which indicates the biocompatibility of the synthesized CePO_4_ NPs over a broad concentration range (Figure 9). This favorable cytocompatibility profile is in accordance with prior biocompatibility assessments of lanthanide-based nanomaterials, which have generally demonstrated low cytotoxicity toward mammalian cells at therapeutically relevant concentrations [46,47].

#### 3.6.2. Results of Investigations on the Effect of CePO_4_ Sols on the Cytotoxicity and Metabolic and Proliferative Activity of Human Keratinocytes

**Figure 10:**
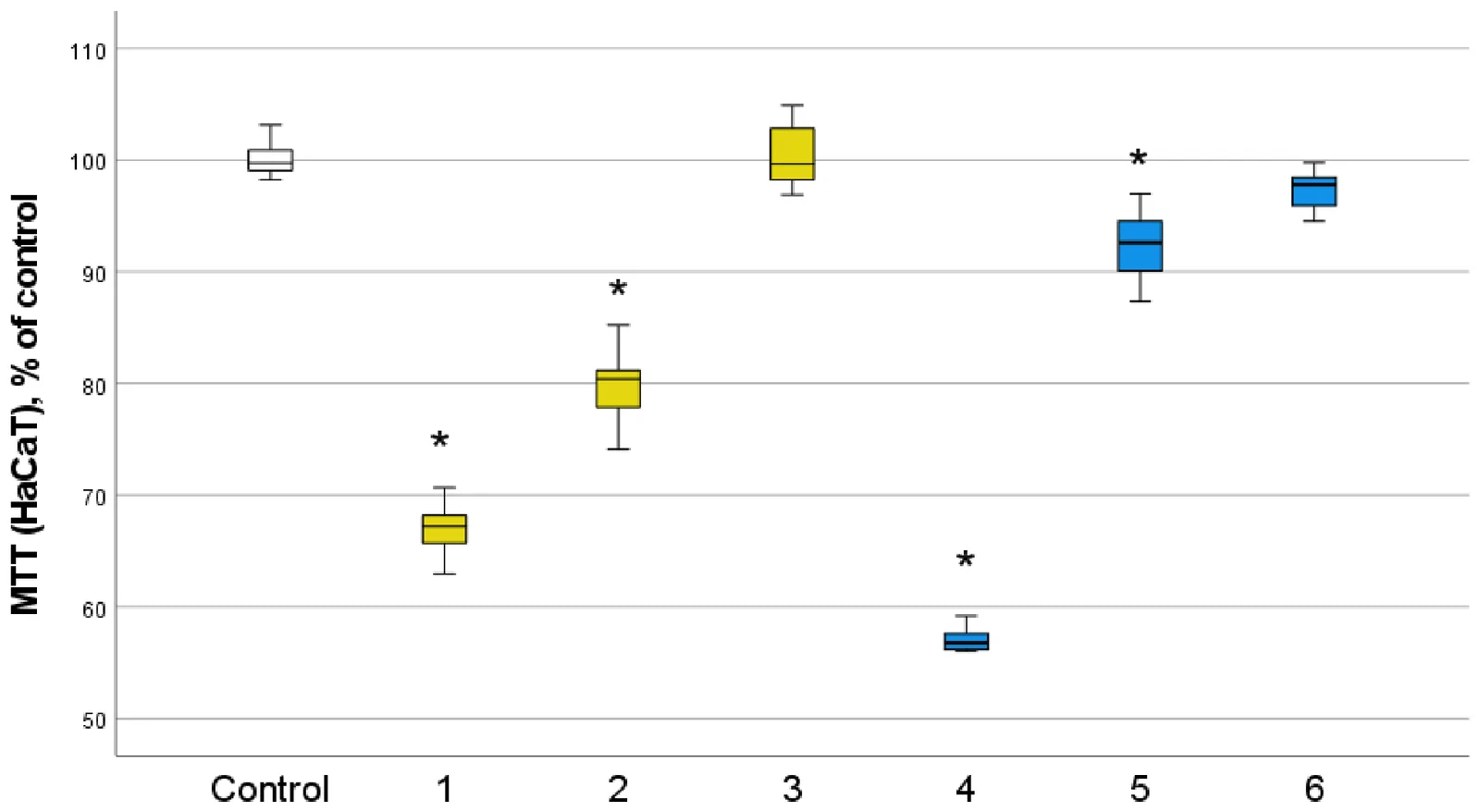
Effect of different synthesis methods and concentrations of cerium phosphate nanoparticles on the metabolic activity of human keratinocytes in the MTT assay. 1—CePO_4_-1 at 10^−2^ M; 2—CePO_4_-1 10^−3^ M; 3—CePO_4_-1 10^−4^ M; 4—CePO_4_-2 at 10^−2^ M; 5—CePO_4_-2 10^−3^ M; 6—CePO_4_-2 10^−4^ M. The Kruskal–Wallis test revealed a statistically significant difference (H = 86.1, df = 6, *p* < 0.001); *—difference from Control at *p* < 0.001.

Keratinocytes proved to be more sensitive to cerium phosphate nanoparticles. The metabolic activity of keratinocytes was significantly suppressed upon co-culture with sols of both nanoparticle synthesis methods at concentrations of 10^−2^ M (the OD value averaged 67.0% and 57.1% for CePO_4_-1 and CePO_4_-2, respectively, relative to the control, *p* < 0.05) and 10^−3^ M (80–92% of the control, *p* < 0.05). Only co-culture with CePO_4_-1 and CePO_4_-2 at a concentration of 10^−4^ M did not affect the metabolic activity of keratinocytes (Figure 10).

**Figure 11:**
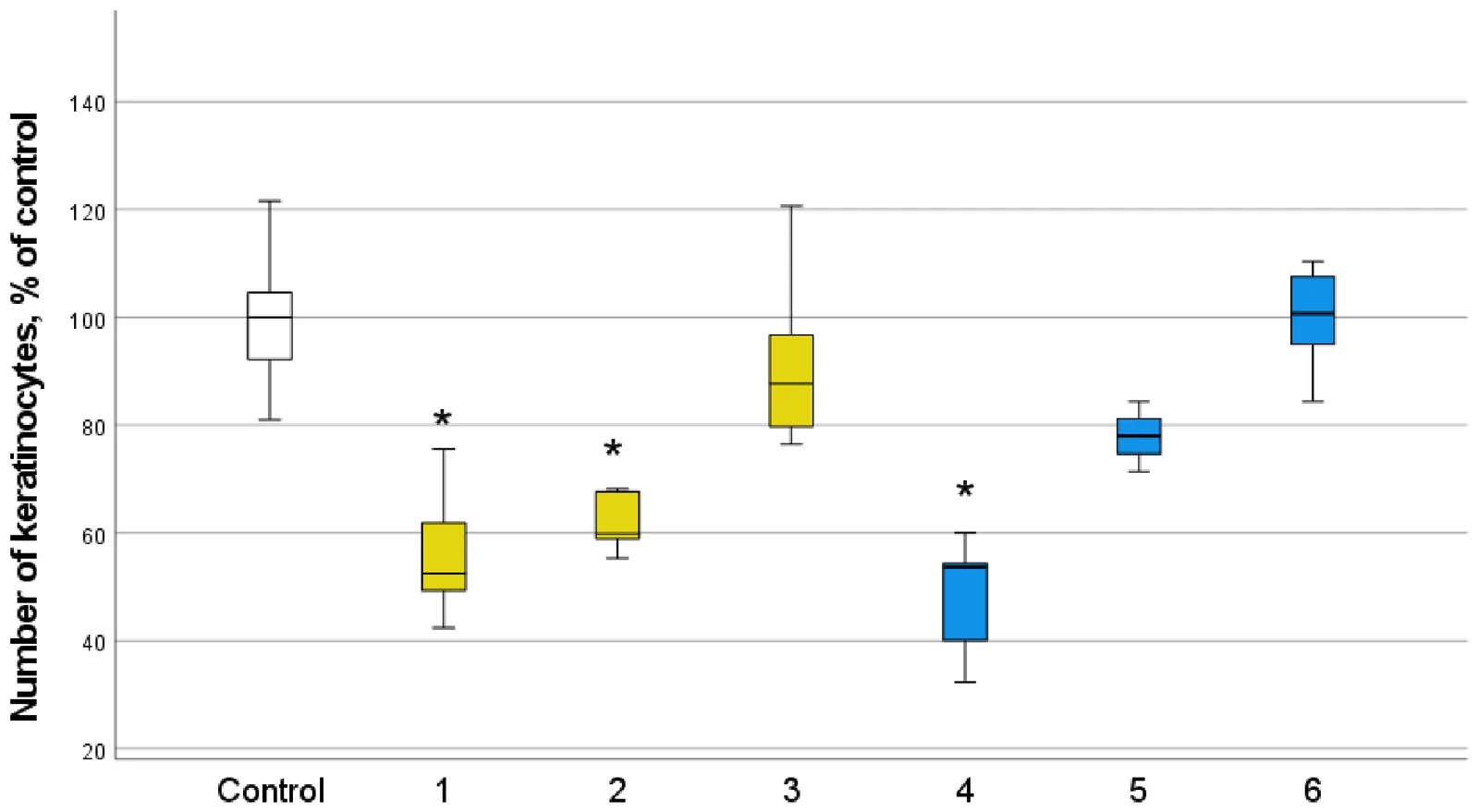
Effect of the concentration and synthesis method of cerium phosphate nanoparticles on the proliferative activity of keratinocytes (HaCaT cell line) as determined by direct cell counting using an automated cell counter. 1—CePO_4_-1 at 10^−2^ M; 2—CePO_4_-1 10^−3^ M; 3—CePO_4_-1 10^−4^ M; 4—CePO_4_-2 at 10^−2^ M; 5—CePO_4_-2 10^−3^ M; 6—CePO_4_-2 10^−4^ M. The Kruskal–Wallis test revealed a statistically significant difference (H = 39.0, df = 6, *p* < 0.001); *—difference from Control at *p* < 0.001.

The proliferative activity of keratinocytes was also suppressed upon co-culture with CePO_4_-1 and CePO_4_-2 nanoparticles at a concentration of 10^−2^ M (keratinocyte counts averaged 54.7% and 47.9% of the control, respectively, *p* < 0.01), as well as with CePO_4_-1 at a concentration of 10^−3^ M (62% of the control, *p* < 0.05). Samples CePO_4_-1 and CePO_4_-2 at a concentration of 10^−4^ M showed no difference from the control group (Figure 11).

**Figure 12:**
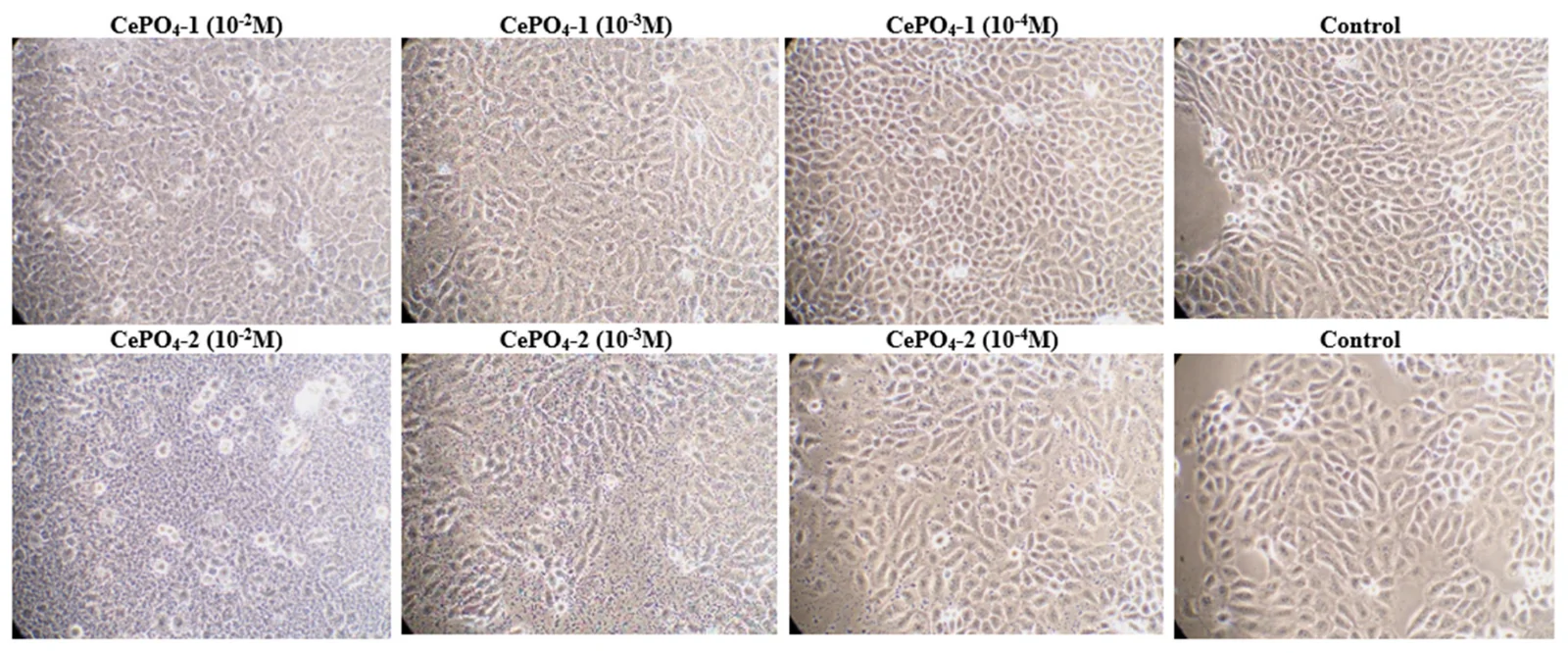
Photographs of keratinocytes (HaCaT cell line) after 72 h of co-cultivation with CePO_4_ of different synthesis modifications and at different concentrations.

Thus, CePO_4_ NPs at a concentration of 10^−4^ M, irrespective of the synthesis method, exerted no effect on human keratinocytes. A suppressive effect of cerium phosphates at concentrations of 10^−2^–10^−3^ M on the proliferation and metabolism of keratinocytes was established. Moreover, the higher the nanoparticle concentration, the greater the degree of suppression of keratinocyte activity. At the same time, no toxic effect of the nanosols on the morphological characteristics of keratinocytes was detected (Figure 12). The differential sensitivity of fibroblasts and keratinocytes to nanoparticle exposure has been previously reported and may be attributed to cell-type-specific differences in endocytic uptake, intracellular trafficking, and redox buffering capacity [48,49].

The percentage of dead cells remained below the detection threshold of the Countess II automated counter in tests with a concentration of 10^−3^–10^−4^ M. The proportion of dead keratinocytes in cocultivation with NPs at a concentration of 10^−2^ M did not exceed 10% and was detected only in occasional (0–1) samples, which supports the biocompatibility of CePO_4_ NPs.

## 4. Discussion

The presented results convincingly demonstrate that the atomic structure of the synthesized cerium orthophosphate nanoparticles is one of the key factors governing their biological activity, thereby opening new perspectives for the development of highly effective wound-healing agents. Synthesis via controlled hydrolysis in the presence of sodium tripolyphosphate enabled the production of two nanomaterials with fundamentally different atomic structures: X-ray amorphous CePO_4_-1, synthesized at 60 °C, and highly crystalline CePO_4_-2 with the rhabdophane structure, formed at 90 °C. These findings are corroborated by both transmission electron microscopy and X-ray diffraction. For CePO_4_-1, micrographs reveal predominantly amorphous agglomerates with a mean particle size of 4.7 ± 1.2 nm, and the diffraction patterns exhibit a halo devoid of distinct Bragg reflections, indicating the absence of long-range order. In contrast, CePO_4_-2 displays well-defined electron diffraction rings and broadened peaks characteristic of the hexagonal rhabdophane phase in the X-ray diffractograms, with an estimated coherent scattering domain size of approximately 7 nm, which is in good agreement with the TEM data (5.2 ± 0.9 nm) and indicates high crystallinity of the individual nanoparticles [50,51].

The enzyme-mimetic bioactivity of the synthesized nanoparticles is substantiated by experimental evidence. XPS confirmed the coexistence of Ce^3+^ and Ce^4+^ on the amorphous CePO_4_-1 surface, providing the redox-active centers required for reversible electron transfer in superoxide dismutase- and catalase-like catalysis. Critically, the significantly higher stimulation of fibroblast metabolism (128%) and proliferation (145%) by defect-rich amorphous CePO_4_-1 compared to crystalline CePO_4_-2, despite identical particle size and chemical composition, cannot be explained by passive material effects and is fully consistent with redox-mediated enzyme-mimetic activity arising from oxygen vacancies.

Analysis of the Ce 3d spectra of the CePO_4_-1 sample revealed the presence of not only satellites characteristic of Ce^3+^ but also low-intensity peaks corresponding to the Ce^4+^ state. This indicates that the amorphized, defect-rich structure promotes the partial oxidation of surface cerium atoms and the formation of a mixed-valence Ce^3+^/Ce^4+^ state [42,52]. The critical role of oxygen vacancies in mediating the catalytic activity of cerium-based nanomaterials has been firmly established through combined experimental and computational investigations, which demonstrate that vacancy-engineered surfaces provide the active sites necessary for efficient ROS scavenging [53,54]. It is precisely the high density of such oxygen vacancies and reversible redox centers, arising from surface hydration-induced disordering, that endows the amorphous nanoparticles with potent enzyme-mimetic activity. This physical mechanism underpins their ability to efficiently catalyze the dismutation of superoxide radicals and the decomposition of hydrogen peroxide, thereby mimicking the functions of superoxide dismutase and catalase [55,56].

The in vitro results fully corroborate this hypothesis: amorphous CePO_4_-1 significantly stimulated both the metabolic activity of fibroblasts (up to 128% relative to the control) and the proliferative activity of fibroblasts (up to 145% relative to the control), effectively protecting them from oxidative stress in an inflammatory phase wound process model, in contrast to its crystalline counterpart [19,57]. The dose-dependent nature of this stimulation, with an optimum observed at intermediate concentrations, is consistent with the hormetic response typically elicited by redox-active nanomaterials, wherein low to moderate levels of catalytic activity promote cellular proliferation, while excessive activity may become inhibitory [58,59]. Our findings on the regenerative advantages of amorphous cerium phosphate over crystalline phosphate are consistent with those obtained by other authors [60]. Amorphous calcium phosphate particles stabilized with inorganic polyphosphate demonstrated high efficacy as an osteoinductive inorganic precursor for new bone formation both in vitro and in vivo. In contrast, crystalline forms of calcium phosphate demonstrated significantly lower regenerative activity.

Notably, the observed divergence at the highest concentration 10^−2^ M—where metabolic activity reaches its maximum while proliferation slightly declines—suggests a metabolic shift. Since the MTT assay reflects the total activity of mitochondrial dehydrogenases, a concentration of 10^−2^ M likely induces a state of metabolic hyperactivation. In this mode, fibroblasts redirect their energetic resources from active division toward cytoprotective mechanisms, cellular maintenance, or the synthesis of extracellular matrix components (e.g., collagen deposition) [61,62,63]. This presumably accounts for the high optical density in the MTT assay, despite a minor, non-significant deceleration in the rate of cell proliferation.

Moreover, the low phonon energy of the CePO_4_ crystalline matrix host, characterized by a maximum lattice vibrational frequency of approximately 1070 cm^−1^, is capable of suppressing non-radiative relaxation, which in the future will enable the fabrication of luminescent scaffolds based on this material for the optical monitoring of dressing biodegradation directly within the wound bed without the use of organic dyes [64,65].

From a physicochemical perspective, the differing stability of the dispersions is also explained by structural features. The small hydrodynamic diameter (7–10 nm), as determined by dynamic light scattering, and the weakly negative zeta potential (approximately −3 mV) promote nanoparticle agglomeration, which is, however, overcome by the introduction of sodium hexametaphosphate [66,67]. By adding a small amount of sodium hexametaphosphate—essentially introducing polyphosphate ions, which are not foreign and readily adsorb onto the particle surface, imparting a negative charge—it is possible to improve the stability of the sols, raising the zeta potential to |30| mV. The advantages of the approach of stabilizing phosphates with phosphate, i.e., without the use of additional elements, have also been highlighted in other studies [35]. The colloidal stability of nanoparticle dispersions is a critical determinant of their biological performance, as aggregation can significantly alter cellular uptake kinetics, biodistribution, and ultimately, therapeutic efficacy [68,69].

UV spectroscopy data, with the characteristic absorption peak of rhabdophane at 273 nm, further confirm the completion of crystalline phase formation at 90 °C. The totality of the obtained data demonstrates that it is precisely the amorphous, defect-saturated structure of cerium orthophosphate, which gives rise to the mixed Ce^3+^/Ce^4+^ valence, that constitutes the physical basis of its antioxidant properties. This provides a clear example of “physical programming,” which we define here as the deliberate control of atomic structure (crystallinity) via synthesis temperature to tune the density of oxygen vacancies and the surface Ce^3+^/Ce^4+^ ratio, without altering chemical composition or nanoparticle size. Through this mechanism, the regenerative activity of the nanomaterial can be programmed at the synthesis stage, enabling the creation of highly effective and biocompatible wound treatment agents that selectively activate fibroblasts and modulate the microenvironment without exhibiting cytotoxicity [70,71]. While this strategy is directly applicable to redox-active rare earth phosphates with variable valence (e.g., Pr, Tb), extension to non-redox-active lanthanides would require doping with redox-active ions (such as Ce) or targeting alternative programmable functions (e.g., luminescence or drug loading).

Since the primary objective of the study was the synthesis of nanoparticles beneficial for enhancing cell proliferation and metabolism, an in vitro investigation using human cell cultures was indispensable. The MTT data obtained from the study of the two synthesized samples on living human cell models demonstrated a significant increase in the proliferative and metabolic activity of fibroblasts, which offers hope for the potential application of cerium phosphate as a regenerative agent in the future. The positive results of this study are consistent with the findings of effective stimulation of human fibroblasts upon co-culture with phosphates synthesized by a different method [20,22,72]. Furthermore, it is noteworthy that the stimulatory effects on human fibroblasts upon co-culture with CePO_4_ NPs and CeO_2_ NPs coincide [20], which may be related to the nature of the principal element of these compounds, cerium. The comparative bioactivity of different cerium compounds underscores the central role of the cerium ion’s redox cycling capability, irrespective of the specific anionic lattice environment [31,73,74].

At the same time, the statistically significant efficacy differed between these samples, correlating with the form and physical characteristics of the obtained compounds. This necessitates the continued search for new methods of cerium nanophosphate synthesis to potentially obtain the most efficacious prototype of a regenerative medical agent. The dose-dependence of the proliferative effect observed in fibroblasts confirms the validity of the conclusions regarding the potentially promising direction for the development of new pharmaceutical agents. However, the relative reduction in the metabolic activity of keratinocyte cell cultures at high concentrations warrants a more thorough investigation of the possible toxic effects of the synthesized cerium nanophosphate compounds. Similar conclusions can be drawn from the analysis of keratinocyte proliferation, where high nanoparticle concentrations further inhibited the activity of this human cell type. This underscores the necessity of conducting a comprehensive suite of instrumental investigations, both in vitro and in vivo, for a well-founded assessment of potential toxic effects.

Thus, the deliberate variation in the synthesis temperature enables the production of both amorphous and crystalline cerium phosphate nanoparticles with virtually identical morphology. Crystallinity and zeta potential stability are not invariably indicative of superior biological activity. In our study, X-ray amorphous CePO_4_-1 (60 °C) was distinguished by a higher capacity to stimulate the metabolism and proliferation of human fibroblasts. At the same time, it is noteworthy that no significant differences were observed when assessing the effect of CePO_4_ NP sols synthesized under different temperature regimes but at identical concentrations, which attests to the biological equivalence of the nanoparticles. The identified biological effects of CePO_4_ NPs, together with their cytocompatibility, may serve as a stimulus for further research and, naturally, upon extensive confirmatory investigations, for the subsequent development of pharmaceuticals for the care of damaged skin and the acceleration of skin wound healing. The translation of lanthanide-based nanomaterials from bench to bedside for wound healing applications will require a multidisciplinary approach encompassing advanced materials synthesis, rigorous preclinical safety assessment, and carefully designed clinical trials [75,76].

### Limitations and Perspectives

The results presented in this article represent the initial steps in evaluating the development of synthesis methods and the potential utility of cerium nanophosphate as a therapeutic agent and, therefore, inevitably have a number of limitations and, correspondingly, perspectives. The study lacks control over the long-term retention of the efficacy of the synthesized prototypes, which must be taken into account given the propensity for aggregation demonstrated in the physical investigations and, consequently, the potential decrease in the desired biological effects during the storage of nanomedicines. Storage conditions for the sols were also not evaluated. Due to the peculiarities of cell culture cultivation capabilities, the study of the biological activity of CePO4 NPs was limited to 72 h in vitro experiments without verifying dissolution behaviour or long-term stability in protein-containing media. A comprehensive suite of toxicological investigations has not been fully conducted on whole living organisms. All of these studies are currently ongoing, and upon their completion and the processing of the obtained information, their results will be presented to the scientific community.

In the present study, we employed cell cultures characteristic of the histological structure of the dermis; therefore, we can currently speak of the regenerative potential of cerium nanophosphate compounds only with respect to their topical application. Nevertheless, this does not preclude the possibility of discovering useful biological potential for other organs and tissues in subsequent investigations. Future studies incorporating 3D skin equivalents, co-culture systems with immune cells, and in vivo wound models will be essential to fully elucidate the therapeutic potential and safety profile of these promising nanomaterials.

## 5. Conclusions

The method of controlled hydrolysis of the complex formed upon the interaction of cerium nitrate and sodium tripolyphosphate enabled the production of aqueous dispersions of hydrated cerium phosphate nanoparticles with a mean size of 4.7–5.2 nm. It was found that synthesis at 60 °C leads to the formation of a predominantly amorphous phase, whereas synthesis at 90 °C yields a predominantly crystalline phase. Notably, with an increase in the synthesis temperature, the mean nanoparticle size increases only marginally, from 4.7 ± 1.2 nm at 60 °C to 5.2 ± 0.9 nm at 90 °C. Thus, the synthesis temperature in this case exerts a greater influence on the crystallinity of the resulting particles than on their size. The resulting hydrated cerium phosphate nanoparticles possess the crystalline structure of rhabdophane, CePO_4_·nH_2_O, as confirmed by X-ray and electron diffraction methods. XPS investigation also indicates the predominant occurrence of cerium in the 3+ charge state within the sample, and the calculated quantitative composition is consistent with the composition of the rhabdophane phase.

It was demonstrated that the synthesis temperature in the range of 60–90 °C is a critical parameter for controlling the phase state of cerium orthophosphate nanoparticles: from an X-ray amorphous phase with elements of short-range order to a fully crystalline hexagonal rhabdophane structure. This distinction has been verified by a combination of TEM, X-ray diffraction, and UV spectroscopy methods, wherein a characteristic excitonic absorption peak at 273 nm was established for the crystalline phase for the first time, and the formation of a mixed-valence Ce^3+^/Ce^4+^ state on the surface of the amorphous nanoparticles was confirmed by XPS.

It was established that it is precisely the amorphous, defect-saturated structure of cerium orthophosphate, characterized by a high density of oxygen vacancies and reversible Ce^3+^/Ce^4+^ redox centers, that constitutes the physical basis of its antioxidant enzyme-mimetic activity. This mechanism directly correlates with biological activity: the amorphous nanoparticles significantly outperform the crystalline ones in their capacity to stimulate the proliferative (up to 144%) and metabolic activity of human fibroblasts, while concurrently exhibiting high biocompatibility. The obtained data prove that the deliberate formation of the amorphous CePO_4_·nH_2_O phase represents an effective strategy for the development of novel wound-healing agents with a redox-mediated mechanism of action.

CePO_4_ nanoparticle sols with a mean diameter of 5 nm, synthesized by different methods, are non-toxic and capable of influencing the biological activity of human cells.

All variants of the synthesized cerium phosphates at all concentrations significantly and dose-dependently activate the metabolic activity of fibroblasts, on average by a factor of 1.14–1.28, with the maximum effect observed at a sol concentration of 10^−2^ M. CePO_4_-1 at concentrations up to 10^−3^ M activates the proliferative activity of fibroblasts (by a factor of 1.45). In keratinocytes, conversely, both metabolic and proliferative activities are suppressed upon co-culture with nanoparticles synthesized by different methods at high concentrations of 10^−2^–10^−3^ M, and only CePO_4_ NPs at a concentration of 10^−4^ M did not affect keratinocyte activity. This leads to the recommendation to use a concentration of 10^−4^ M, especially for superficial skin wounds.

The absence of significant differences in experiments on human cells when assessing the effect of identical concentrations of CePO_4_ NPs synthesized under different temperature regimes demonstrates the biological equivalence of the nanoparticles.

The observed biological effect supports the recommendation for further investigations of the nanoparticles, including their application in the development of preparations that accelerate regeneration and the treatment of skin wounds.

## Data Availability

The original contributions presented in this study are included in the article. Further inquiries can be directed to the corresponding author.
